# Self-Resolving Mobitz Type II Second-Degree Heart Block (Atypical Wenckebach Block) After Cesarean Section Under Subarachnoid Block: A Case Report

**DOI:** 10.7759/cureus.10704

**Published:** 2020-09-29

**Authors:** Pratap Rudra Mahanty, Abhishek Chatterjee, Deb Sanjay Nag, Rajiv Shukla

**Affiliations:** 1 Anaesthesiology, Tata Main Hospital, Jamshedpur, IND

**Keywords:** caesarean, arrhythmias, heart block

## Abstract

The majority of the perioperative arrhythmias in patients undergoing cesarean section under spinal anesthesia are benign. We report a case of a 30-year-old full-term parturient with a history of an uneventful previous cesarean section. She had no preexisting comorbidities. She subsequently underwent another emergency cesarean section three years later due to abdominal pain and scar tenderness indicative of impending rupture. Two hours after an uneventful surgery, the patient developed epigastric pain with a prolonged PR interval (280 ms) and intermittent second-degree AV block with two consecutive blocked P waves, which was consistent with Mobitz type II second-degree heart block (atypical Wenckebach block). However, she remained hemodynamically stable throughout. Serial electrocardiogram (ECG) did not demonstrate any evidence of ST-T wave changes, and normal troponin I and echocardiography excluded myocardial ischemia as a potential cause for the arrhythmia. Normal serum electrolytes and the resolution of the sensorimotor block caused by the spinal anesthesia excluded other known causes for such ECG changes. The PR interval gradually decreased to 240 ms on the second postoperative day and normalized to 200 ms on the fifth postoperative day. Such patients, especially those with a wide QRS complex, are susceptible to developing dangerous ventricular arrhythmias that can adversely affect circulatory function. Close vigil is the key to avoiding adverse perioperative outcomes.

## Introduction

The incidence of perioperative arrhythmias in parturients undergoing cesarean section is higher than expected. While most arrhythmias are transient and innocuous, they are often unexpected and may need treatment. Those with severe bradycardias, multiple ventricular premature complexes (VPCs), and atrioventricular (AV) blocks are at higher risk of developing severe, dangerous ventricular arrhythmias that can adversely affect circulatory function [1]. Close vigil and the identification of perioperative rhythm abnormalities are key to avoid adverse perioperative outcomes.

## Case presentation

A 30-year-old full-term parturient without any comorbidity and history of previous cesarean section three years ago was admitted for emergency cesarean section due to abdominal pain and scar tenderness indicative of impending rupture. A pre-anesthesia evaluation did not reveal any abnormality. The preoperative ECG showed normal sinus rhythm with a PR interval of 0.18s. A subarachnoid block was administered at the L3-L4 space with intrathecal 2.2 ml of 0.5% hyperbaric bupivacaine. An assessment of sensory block height using touch and cold demonstrated a sensory block up to the T4 level; motor blockade by the modified Bromage scale was Grade 3 [2]. Intraoperative blood pressure was maintained within 10%-20% of the baseline values with aliquots of 3-6 mg intravenous ephedrine. There was no episode of brady or tachyarrhythmias intraoperatively. After the delivery of the baby, she received intravenous oxytocin 5 IU over five minutes to facilitate the third stage of labor. Postoperative analgesia was managed with intravenous paracetamol and intramuscular diclofenac sodium. Intravenous fentanyl was used as a rescue analgesic. No other drug was administered in the perioperative period.

**Figure 1:**
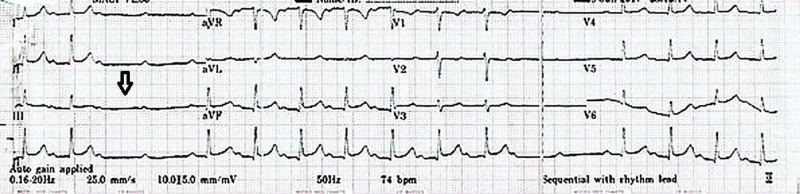
Intermittent second-degree AV block with two consecutive blocked P waves AV: atrioventricular

After an uneventful surgery, the patient was shifted to the post-anesthesia care unit, where she started complaining of epigastric pain after two hours. The patient had only mild pain on movement at the operative site with a visual analog scale (VAS) score of 2. An irregular pulse was noted on examination, and the cardiac monitor showed an irregular ECG pattern with occasional blocked P-waves. There was no hemodynamic instability. A 12-lead ECG was urgently conducted showed a prolonged PR interval (280 ms) and intermittent second-degree AV block with two consecutive blocked P waves (Figure 1).

**Figure 2:**
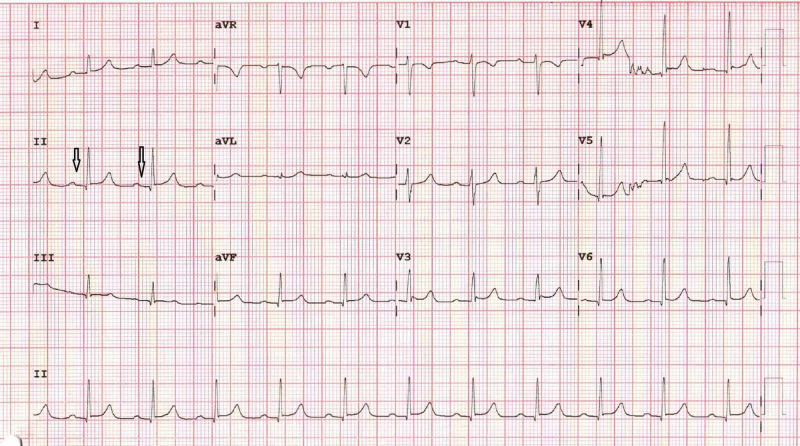
Normal PR interval

The arterial blood gas (ABG) and other routine blood investigations were within normal limits. As the patient continued to remain hemodynamically stable throughout, no pharmacologic intervention was needed. Troponin I measured at six-hour intervals was within normal limits. A serial 12-lead ECG revealed no significant ST-T wave changes. Echocardiography also did not reveal any abnormalities. The patient’s epigastric pain gradually resolved, and the patient was comfortable by the second postoperative day. An ECG on the second postoperative day revealed a PR interval of 240 ms with a normal QRS complex and sinus rhythm. The patient's medication chart was reviewed and the possible administration of any concomitant drug with a deleterious arrhythmogenic effect was excluded. The patient was discharged from the hospital on the fifth postoperative day with sinus rhythm and a PR interval of 200 ms (Figure 2). No antiarrhythmic drug was prescribed in the postoperative period.

## Discussion

Among the various causes of perioperative arrhythmias in patients undergoing cesarean section under spinal anesthesia, the maternal causes include hemodynamic alterations or hypokalemia of pregnancy, hormonal changes during pregnancy, or autonomic dysregulation. A subarachnoid block can cause cardiac autonomic balance shifts towards parasympathetic overactivity due to pharmacologically achieved sympatholysis [3]. Exteriorization of the uterus during the surgery can also result in vagus nerve-mediated cardio depressor reflex such as Bezold-Jarisch reflex (BJR) [3]. However, our patient demonstrated no ECG abnormality in her preoperative evaluation and remained hemodynamically stable throughout her intraoperative period. A sensory block of up to T4 after the subarachnoid block and the development of symptoms two hours postoperatively when the sensory loss had regressed and the motor block of the lower limbs had waned to Grade 0 on a modified Bromage scale excluded a high subarachnoid block as a potential cause for the symptoms. Myocardial ischemia is a known cause of second-degree AV block. Normal troponin I levels, no significant ST-T wave changes on serial ECG, and normal echocardiography excluded myocardial ischemia as the potential cause.

Different variants of conduction defects, including type 1 or type 2 heart blocks, have been associated with a subarachnoid block (SAB) in parturients. Shen et al. studied 254 healthy parturients who received SAB and observed that the incidences of both type 1 and type 2 heart block were 3.5% [1]. An ECG in our case showed the appearance of P waves at regular intervals with prolonged PR duration (280 ms) and two consecutive blocked P waves. This is consistent with a Mobitz type II second-degree heart block (atypical Wenckebach block). A typical Wenckebach block is considered when the first PR interval of a cycle is the shortest, and there is a progressive lengthening of the PR interval with the increment between the first and second conducted beats being the largest, followed by a progressive decrease in the RR intervals [4]. Narula and Samet reported that a conduction block in the His bundle or lower in the conduction system results in Mobitz type II second-degree AV block [5]. If this block is associated with a wide QRS complex, these patients have a greater risk of mortality, and definite measures, such as pacemaker insertion, are needed to stop the further progression of the AV block [5]. Similarly, Smith et al. concluded that a Mobitz type II second-degree AV block, when associated with wide QRS complex, indicates diffuse conduction system disease and is an indication for pacing, as it may degenerate into third-degree heart block [6]. 

In our case, the QRS complex remained normal without any manifestation of pre-syncope or syncope. Therefore, no interventions were required. Kalra and Hayaran reported similar findings [7]. However, Kalra and Hayaran reported an episode of bradycardia during surgery, which resolved spontaneously without any intervention. Mangiardi et al. concluded that atropine increases the sinus rate significantly and causes minimal improvement in AV nodal conduction [8]. However, because of atrial tachycardia, the block may further worsen and, hence, atropine is contraindicated in such conditions [8]. Our patient did not have any episode of bradycardia during the perioperative period and no pharmacological interventions were needed. Normal serum electrolytes excluded dyselectrolytemia as the causative factor. Greene and Brull postulated that following SAB, a sympathetic block is slower to regress [9] and considerable sympathetic blockade can persist in the postoperative period. However, considering the limited duration of action of intrathecal bupivacaine and the gradual resolution of the Mobitz type II second-degree heart block over five days makes this an unlikely cause [10]. A self-resolving Mobitz type II second-degree heart block (atypical Wenckebach block) with an increased PR interval normalizing over five days after a cesarean section under SAB has not been reported until now.

Patients with severe bradycardias, multiple ventricular premature complexes (VPCs), and AV blocks in the perioperative period are at a higher risk of developing dangerous ventricular arrhythmias, which can adversely affect circulatory function. In severe cases, cardioversion or pacing has to be considered. In our case, as a significant hemodynamic compromise was not evident, we maintained closed monitoring, keeping preparations for other interventions, should the need arise. Therefore, through this report, we wish to highlight the importance of close vigil of parturients undergoing a cesarean section, not only in their intraoperative period but also in the postoperative period too, in order to prevent adverse outcomes. 

## Conclusions

Arrhythmias are the most common cardiac complications encountered during pregnancy. Its incidence is higher than expected. Some patients, especially those with a wide QRS complex, are susceptible to developing dangerous ventricular arrhythmias, which can adversely affect circulatory function. Close vigil during the entire perioperative period is key to avoid adverse perioperative outcomes.
